# Supplementary material for: Predicting visual function by interpreting a neuronal wiring diagram
Source: Nature. 2024 Oct 2;634(8032):113–23. doi: 10.1038/s41586-024-07953-5 (PMC11446822; doi:10.1038/s41586-024-07953-5)
Supplement: Supplementary file 6 — CRF and ERF predictions for individual TmY4 and TmY9 cells. Analogous to Supplementary Data 3, but for TmY target types. Shown are the top four monosynaptic pathways, the strongest pathway passing through each of the top ten intermediary types (ranking from Extended Data Fig. 7), and the trisynaptic pathway Tm1–TmY–Dm3–TmY (see the section entitled Prediction of spatial normalization). [file 41586_2024_7953_MOESM6_ESM.zip › DataS4/TmY9qΓèÑ/720575940646768163.pdf]

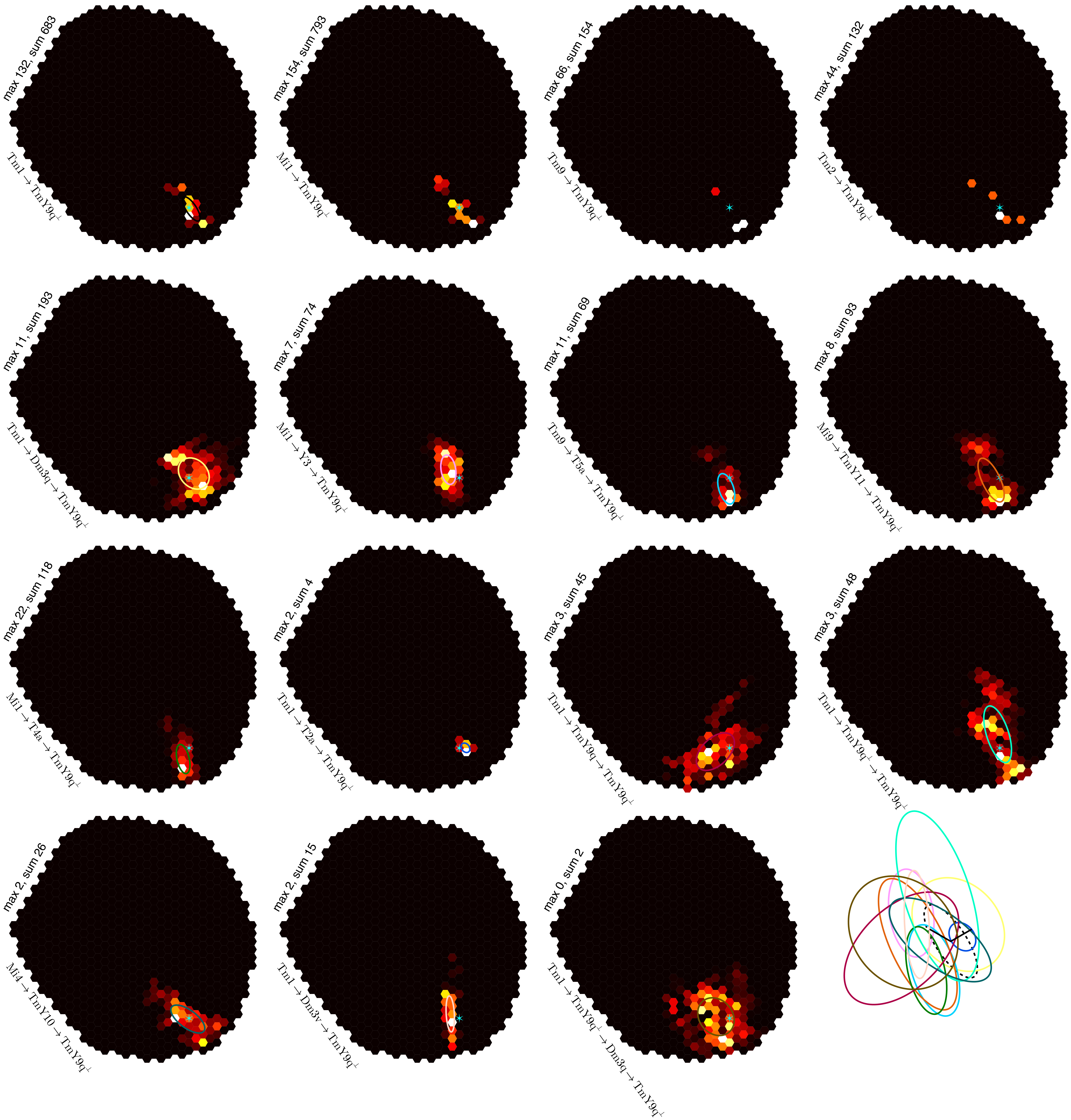

max 132, sum 683
Tm1 → TmY9q⊥
max 154, sum 793
Mi1 → TmY9q⊥
max 66, sum 154
Tm9 → TmY9q⊥
max 44, sum 132
Tm2 → TmY9q⊥
max 11, sum 193
Tm1 → Dm3q → TmY9q⊥
max 7, sum 74
Mi1 → Y3 → TmY9q⊥
max 11, sum 69
Tm9 → T5a → TmY9q⊥
max 8, sum 93
Mi9 → TmY11 → TmY9q⊥
max 22, sum 118
Mi1 → T4a → TmY9q⊥
max 2, sum 4
Tm1 → T2a → TmY9q⊥
max 3, sum 45
Tm1 → TmY9q → TmY9q⊥
max 3, sum 48
Tm1 → TmY9q⊥ → TmY9q⊥
max 2, sum 26
Mi4 → TmY10 → TmY9q⊥
max 2, sum 15
Tm1 → Dm3v → TmY9q⊥
max 0, sum 2
Tm1 → TmY9q⊥ → Dm3q → TmY9q⊥
